# Exploring Actinobacteria for new insecticides and their delivery in crop protection

**DOI:** 10.1099/mic.0.001690

**Published:** 2026-03-24

**Authors:** Lachlan Dow, Louise F. Thatcher, Joshua Porter, Anna Marcora, Alexandra Gloria, Marta Gallart

**Affiliations:** 1 Commonwealth Scientific and Industrial Research Organisation (CSIRO) Agriculture and Food, Acton, ACT, Australia; 2 Commonwealth Scientific and Industrial Research Organisation (CSIRO) Microbiomes for One Systems Health Future Science, Canberra, Australia; 3 Commonwealth Scientific and Industrial Research Organisation (CSIRO) Advanced Engineering Biology Future Science Platform, Acton, ACT, Australia

**Keywords:** *Actinobacteria*, bioinsecticide, crop protection, insecticide, natural products

## Abstract

Crop protection is essential for agricultural production systems, safeguarding yields and product quality. Chemical controls are a mainstay of protection; however, regulatory and consumer demands, environmental concerns and a general overreliance resulting in resistance development in pest populations have led to increased interest in biopesticides and environmentally friendly alternatives. Biopesticides targeting insects include micro-organisms and their derivatives, such as peptides and specialized metabolites. Their target specificity, structural complexity, modes of action and environmental safety are key differentiators to chemical controls, and when used in integrated pest management programmes, biopesticides can reduce reliance on chemical pesticides and promote sustainable agriculture. As the demand for bioinsecticides grows, so too has the research and application of micro-organisms, alongside their taxonomic diversity and isolation sources. Of key interest are *Actinobacteria* as both promising and well-tested alternatives for managing insect pests in various agricultural settings, with several products commercialized for use across a variety of crops and target pests. Recent advances and investigations in metabolomics and genomics highlight the untapped and significant biochemical potential and value of *Actinobacteria* for natural product discovery. This review covers a broad spectrum of published literature that has reported on insecticidal biological activity data associated with *Actinobacteria* or their natural products. We also report on *Actinobacteria*-derived nematicides and acaricides that are significant for crop protection. The origin of these natural products, their structural diversity and notable substructures are discussed, along with new areas for discovery and avenues for enhancing screening methods and metabolo-genomics approaches.

## Introduction

Arthropod pests are responsible for significant costs in global food and fibre production: in the order of billions of dollars each year across protected and unprotected cropping systems [1]. This can be through direct damage of plant tissues, indirect transmission of viral plant diseases or the emergence of insecticide resistance. Chemical insecticides, defined as pesticides that are formulated to kill, harm, repel or mitigate one or more species of insect [2], are the predominant treatment to protect crops from these deleterious effects. However, the effectiveness and availability of chemical insecticides are constrained by insect resistance to many insecticide products due to limited modes of action, effects on non-target species, undesirable environmental impacts, reduced market access and growing concerns amongst growers and consumers, along with increasing regulatory requirements [3]. There has been a deceleration in the discovery pipeline for new synthetic chemistries, with the last major class (diamides) introduced in 2008 [4,5], while the cost and time to discover and bring a new insecticide to market have steadily increased since the 1980s [3,6, 7]. These limitations highlight the need for the discovery and development of novel, effective insecticidal agents with new modes of action and reduced off-target impacts on the environment [8,9].

Biopesticides are a diverse group of pest control products based on naturally occurring microbes, biochemicals or minerals [10,12]. Microbe-based biopesticides contain living micro-organisms, or their by-products or derivatives, as their main active ingredient. The effects and modes of action of a biopesticide depend on the active ingredient, method of application and targeted pest. Bioinsecticides are one class of biopesticides. They may act rapidly or be slow to kill, inhibit insect feeding, act as repellent or oviposition deterrent or exhibit insect growth regulatory effects [13,14]. Insect suppression or insecticidal effects can be through direct or indirect mechanisms. Some microbial bioinsecticides directly kill or inhibit the insect through parasitism, toxins or antibiosis and the production of inhibitory compounds that limit or reduce insect activity, growth or reproduction. Bioinsecticides that work through indirect mechanisms do so by creating conditions unfavourable for insect survival. This includes, for example, the priming or induction of plant resistance mechanisms [15].

Many microbes, as well as plants and various marine and terrestrial organisms, produce or biosynthesize a diverse range of biologically and structurally varied natural products (NPs), such as specialized metabolites, peptides and enzymes. These compounds enhance their overall fitness and survival prospects by acting as an evolutionary defence against abiotic and biotic pressures [16]. The highly diverse metabolic potential and biological roles often reflect an organism’s ability to survive in different environments. A generalist microbe may have evolved to possess a rich metabolic diversity that facilitates its growth across multiple environments, while a microbe adapted to a niche environment with a specialist lifestyle would require less extensive metabolism but more specific adaptations to the conditions within that environment [17]. An example of the former is the *Actinobacteria* (*Actinomycetota*) phylum. Species within the *Actinobacteria* phylum display vast and versatile metabolic potential, as evidenced by their wide distribution across terrestrial and aquatic environments, as well as within the microbiomes of plants and animals [17,18]. Many *Actinobacteria* species produce chemically diverse specialized metabolites that exhibit bioactivities such as insecticidal, bactericidal, acaricidal, herbicidal, fungicidal and plant growth-promoting effects, as well as stimulating plant immunity [18,21]. These organisms and the metabolites they produce can be a source for discovering new bioinsecticides.

The primary aims of this review are to highlight the current landscape in microbe-based and microbially derived bioinsecticides, with a specific focus on the *Actinobacteria* phylum and opportunities for novel bioinsecticide discovery, improvement and deployment in agriculture for crop protection. We highlight fermentation extracts derived from *Actinobacteria*, as well as NP structures and classes, including specialized metabolites and peptides, that have demonstrated significant insecticidal bioactivity in laboratory, glasshouse or field studies. We extend this to include those with nematicidal or acaricidal activity and potential for insect control. We note their producer strains, associated targets and mode of action (MoA) if known, comparing bioactivity and structural diversity amongst related structures to identify potential areas for new NP discovery. This information will aid in the pre-commercial assessment of active structures identified from novel strains, determining their viability for potential commercialization, as well as identifying those already commercialized or not viable. We detail cases where semi-synthetic strategies have overcome stability or efficacy constraints, facilitating the commercialization of bioinsecticide NPs. Finally, we explore opportunities using metabolo-genomics integrated approaches, bioactivity screening and novel insecticide discovery.

### Drivers for change

Insecticides of the past were largely dominated by chlorinated hydrocarbons, methylcarbamates and organophosphates [22]. Many compounds within these categories have since become less effective as resistant pest populations emerged and are now less commonly used due to widespread restrictions imposed because of their environmental persistence and toxicity [23]. The more recent neonicotinoid class of insecticides, which are comparatively less toxic to humans, is now the most widely used insecticide globally, accounting for 25% of all pesticides used [24]. Even with insecticides and their increasing use over time, crop losses to insect pests have not decreased [25]. Overall losses are particularly high in crops grown under high productivity conditions, such as large-scale monocultures of genetically uniform crops, reduced crop rotation and/or reduced tillage. Widespread prophylactic use of some insecticides, their inappropriate or excessive use, can lead to resistance and the inadvertent destruction of pollinators or natural insect pest enemies.

With the increasing use of single MoA products comes the development of insecticide resistance and associated challenges for sustainable crop production. While there are over 25 chemical MoAs available to control insect pests, this is not huge considering the diversity of pest species around the world. Over the past 50 years, there has been an increase in the number of cases of insecticide resistance, as well as the species and compounds involved [3,5]. More than 600 species of insects and related arthropods are now resistant to insecticides [3]. Furthermore, several insect pest species have developed resistance to multiple insecticides. This phenomenon is partly associated with agronomic practices, the global range of crops and intensification, alongside factors related to pest biology and genetics [26]. Insecticide resistance combined with toxicity, regulatory or market-access restrictions, consumer concerns and increased scrutiny on the use of insecticides in sensitive environmental areas is steering us towards a future with limited chemical options and driving the adoption of new interventions for insect control, such as bioinsecticides [27,28]. This is particularly evident in the case of neonicotinoids, the use of which was severely restricted or banned for outdoor use in the EU, less than 10 years after their initial approval, due to off-target effects [29]. Consequently, there is significant interest and growth in the discovery, development, commercialization and uptake of bioinsecticides on farms [30].

### Mitigating insecticide resistance with bioinsecticide modes of action

Bioinsecticides, in contrast to many chemical insecticides, typically target pests through modes of action that are different from those of synthetic options, and they often exhibit a greater diversity of modes of action. Furthermore, those applied as microbial inoculants and/or in the form of fermentation mixtures may also act through multiple modes of action.

As a result, bioinsecticides can play a crucial role in managing insecticide resistance by offering alternative control methods and reducing the selection pressure for resistance, thereby contributing to sustainable pest control [31,33]. An example of how the adoption of biologicals has addressed a resistance issue is a progressive integrated pest management (IPM) approach implemented by the Australian cotton industry to control the cotton bollworm (*Helicoverpa armigera*). A reliance on and intense use of non-selective insecticides led to rapid resistance. Effective IPM was facilitated through farmer education and IPM advisory services, cultivation of Bt cotton (genetically modified cotton plants that express a natural toxin from the soil bacterium *Bacillus thuringiensis*) and availability of selective insecticides, including two *Actinobacteria*-derived NP (spinosad and emamectin, described further in the following sections) that provided effective control with less impact on beneficial insect populations [34].

The international Insecticide Resistance Action Committee (IRAC) is focused on ensuring the long-term efficacy of insect, mite and nematode control products, including biopesticides, through effective and sustainable resistance management [5]. The IRAC MoA classification scheme categorizes insecticides based on their MoA [35]. Most commercially valuable chemical insecticides have been neuro-active agents, with the four major classes and associated MoA including the acetylcholinesterase inhibitor carbamates and organophosphates (group 1), the gamma-aminobutyric acid (GABA)-gated chloride (GluCl) channel blocker organochlorines (group 2), the sodium channel modulator pyrethroids and dichlorodiphenyltrichloroethane or DDT (group 3) and the nicotinic acetylcholine receptor (nAChR) competitive modulator neonicotinoids (group 4) [23,36Casida, 2015, Li et al., 2020]. The *Actinobacteria*-derived NP spinosyns and avermectins are classified as nAChR allosteric modulators–site 1 (group 5) and GluCl allosteric modulators (group 6), respectively. Reflecting the increasing number of microbe-based products, new MoA groups have been added to the IRAC classification scheme. These include species of the bacterium *Bacillus*, including *Bacillus thuringiensis* (*Bt*; group 11), baculoviruses (group 31) and a peptide-based insecticide (group 32). As the MoA of most microbial biopesticides has yet to be identified, they are categorized into four broad groups under a section titled *unknown* or *undefined MoA*. This includes UNB or unknown non-*Bt* bacterial agents, e.g. *Burkholderia* species, UNE or botanical essence, including synthetic extracts and undefined oils, such as neem oil, UNF or fungal agents, such as *Beauveria bassiana* strains, and UNM or non-specific mechanical agents, such as diatomaceous earth [26].

### Restricted diversity in current commercialized bioinsecticides

The microbe-based bioinsecticide market is dominated by products of *Bacillus thuringiensis* and entomopathogenic fungi, such as *Beauveria bassiana* and *Metarhizium anisopliae* [37,38]. The soil-dwelling, spore-forming bacterium *Bacillus thuringiensis* (*Bt*) produces insecticidal proteins that are effective against insect larvae, particularly caterpillars. There are hundreds of subspecies or strains of *Bt*, with some widely used in commercial bioinsecticides. The first practical application of *Bt* for pest control was in the 1920s, followed by the first commercial product, Sporine, introduced in France in 1938 [39]. *Bt* can be considered the most successful bioinsecticide during the last century [40], dominating the bioinsecticide market and accounting for 90% of commercial microbial bioinsecticides [41]. *Bt* strains produce crystal (Cry) or cytolytic (Cyt) proteins during sporulation or vegetative insecticidal proteins (Vip) during vegetative growth, which are toxic to specific insect larvae when ingested. *Bt*-based products are highly specific, safe, widely used and have shown high effectiveness. However, their effectiveness is restricted to specific insect species within the Coleoptera, Diptera and Lepidoptera families.

*Beauveria bassiana* is a widely distributed entomopathogenic fungus that is extensively used as a bioinsecticide. It targets a variety of insects, having a broad host range and the ability to parasitize over 700 different insect species, including whiteflies, aphids and thrips [42]. *Metarhizium anisopliae* is another entomopathogenic fungus. It targets pests like termites, beetles and locusts and has been used in agricultural and urban environments to manage these pests. As this bioinsecticide requires the germination of fungal spores and their penetration of the insect cuticle, it may not kill the insect pest population immediately. However, it will gradually reduce the population in the weeks following application. Other fungal-based bioinsecticide products include *Lecanicillium lecanii* (formerly known as *Verticillium lecanii*) and *Isaria* sp*.* (formerly *Paecilomyces* sp*.*). These bioinsecticides parasitize and kill their host using hydrolytic enzymes and toxic NPs [43]. Comparative studies of *Lecanicillium lecanii* conidial formulations vs. filtrates from fermentation extracts demonstrated that the filtrates had a higher mortality rate against whiteflies (*Bemisia tabaci*) and aphids (*Myzus persicae*) (41% and 62%, respectively), compared to the conidia (19% and 54%), highlighting the potency of the secreted NPs [44].

In addition to the plethora of *Bacillus*- and fungal-based products, *Actinobacteria*-derived bioinsecticides have gained attention for their effectiveness in targeting insect pests [19,45, 46]. Three compounds that exemplify the successful exploitation of *Actinobacteria* NPs for commercial agricultural purposes are the polyketide insecticides spinosad, produced by the strain *Saccharopolyspora spinosa*, avermectins from *Streptomyces avermitilis* [47,48] and milbemycins from *Streptomyces hygroscopicus* (Table 1). Spinosad is effective against a wide range of insect pests, including caterpillars, thrips and leafminers [49]. It works by causing hyperexcitation of the insect’s nervous system, leading to paralysis and death [50]. Avermectins are effective against mites, leafminers and other insect pests where they interfere with the nervous system of insects, causing paralysis and death [51]. Structurally similar to avermectins, milbemycins disrupt the nervous system of insects. They are effective against mites and other arthropods [49]. We discuss these NPs and other bioinsecticides derived from *Actinobacteria* in detail in the ‘Insecticidal natural products from *Actinobacteria*’ section.

**Table 1.** Examples of commercialized microbial and microbially-derived bioinsecticide products Note, the list of microbial species and commercialized products is not exhaustive.

Microbial species	Target pest	Product – microbe, fermentation solids, compound	Examples of commercial product	IRAC group and bioactive compound if known
*Bacillus thuringiensis/ Bacillus sphaericus*	Insect larvae (caterpillars, mosquitoes)	Microbe (bacteria), NP	Various; Dipel, Bollgard II, XenTari, Delfin, Agree, Campbell, Costar, Vectolex, Thuricide, VectoBac	11; *Bt*
*Beauveria bassiana*	Primarily used for controlling Hemiptera, Lepidoptera and Coleoptera pests, e.g. whiteflies, aphids, thrips, ants, fall armyworm	Microbe (fungus)	Various; Broadband, Velifer, BotaniGard, Mycotrol	UNF
*Burkholderia rinojensis*	A broad range of sucking and chewing insects, mites and flies	Microbe (dead bacteria)	Venerate	UNB
*Chromobacterium subtsugae*	Mealy bugs, mites, thrips, aphids	Fermentation solids (dead cells+compounds)	Grandevo	NC
*Isaria fumosorosea/Isaria lilacinus* (formerly *Paecilomyces*)	Whiteflies, nematodes	Microbe (fungus)	PreFeRal, MeloCon, BioAct	UNF
*Lecanicillium lecanii/Lecanicillium muscarium* (formerly *Verticillium*)	Whiteflies, aphids, thrips, mealy bug	Microbe (fungus)	Mycotol and Vertalec	UNF
*Metarhizium anisopliae*	Termites, beetles, locusts, grasshoppers	Microbe (fungus)	Various; Green Guard, Met52, BioCane,	UN
Polyhedrosis virus of *Helicoverpa zea*	*Helicoverpa* spp. (bollworm)	Virus	Gemstar	UNV
*Saccharopolyspora spinosa*	Caterpillars, thrips, psyllid	Compound	Success Neo, Radiant	5; spinetoram
*Saccharopolyspora spinosa*	Lepidopterous larvae (worms or caterpillars), leafminers, thrips, fruit fly	Compound	Naturalure, Entrust, Tracer	5; spinosad
*Streptomyces aureus*	Mites	Compound	Mitecidin, Mitedown	Polynactins
*Streptomyces avermitilis*	Mites, leafminers, leafhoppers, thrips, nematodes	Compound	VertimecPro, Agri-Mek/Agrimec, Tervigo, Avicta	6; abamectin
*Streptomyces hygroscopicus*	Mites	Compound	Milbeknock	6; milbemycin
*Streptomyces lydicus*	Registered biofungicide (can aid in control of nematodes)	Microbe (bacteria)	Actinovate	–

Source: [14,39, 42, 53, 115, 124, 125].

## *Actinobacteria*-based biopesticides

There is considerable ongoing interest in *Actinobacteria* NPs and their producer strains as microbial inoculants for controlling insect pests and plant diseases in agriculture. *Actinobacteria* are Gram-positive bacteria known for their high GC content and complex life cycles, with many exhibiting a mycelial lifestyle and intricate morphological differentiation [18]. They have been isolated from diverse environments, including soils, rhizospheres, as endophytes, and from the gastrointestinal tracts of insects and animals, as well as from freshwater and saline environments, thermal vents and marine sponges [18,52]. These bacteria thrive in a wide range of diverse and sometimes extreme environments, often dominating the microbiota of macro-organisms. While many exhibit slow growth rates, they possess highly resilient spores and specialized metabolism, resulting in the remarkable production of diverse NPs. These NPs play critical roles for their producer strains in various ecological processes, such as interactions with macroscopic plants and invertebrates, and in their survival, serving to outcompete faster-growing microbes. *Actinobacteria* represent an almost limitless source of bioactive NPs and are amongst the most species-rich and diverse bacterial phyla identified [52,53]. Scientific studies have led to numerous discoveries and the exploitation of these compounds, particularly in pharmaceutical applications, but also across agricultural industries [18,19, 22, 45, 52,54]. This has seen many *Actinobacteria* NPs commercialized for crop protection (Table 1). By contrast, fewer products based on *Actinobacteria* microbial inoculants have been commercialized [19,20].

*Actinobacteria* are reported to produce more than 10,000 NPs, with the potential for even greater diversity and production [33]. This includes additional metabolic potential identified in strains that have been extensively studied in the past [17]. While one of the most well-known genera, *Streptomyces*, is notable for its ability to produce nearly two-thirds of known antibiotics [18], ~20–30% of the 10,000 NPs produced by *Actinobacteria* originate from non-*Streptomyces* genera, such as *Micromonospora*, *Microbispora*, *Norcadia* and *Saccharomonospora* [55]. In the following sections, we identify and highlight the diversity and use of *Actinobacteria* strains or their derivatives as biopesticides, covering both academic research and commercial applications.

### *Actinobacteria* inoculants or extracts with bioinsecticide activity

Amongst the *Actinobacteria*, *Streptomyces* are the most extensively studied in agricultural settings. This is attributed to their frequent isolation from these environments, traits that are beneficial to plants, their relatively rapid growth and well-defined small- and large-scale fermentation conditions. As of March 2025, a literature search for *Actinobacteria* delivered as ferment broths, filtered ferments (supernatant) or solvent extracts from cultures for the control of insect pests identified only studies reporting activity from *Streptomyces* species. There are multiple examples showing evidence of *Streptomyces* ferment broths or solvent extracts exhibiting activity against the Lepidoptera larvae *Helicoverpa armigera* (Cotton bollworm), *Spodoptera littoralis* (Cotton leaf worm) and *Plutella xylostella* (Diamondback moth) [56,59]. This included *Streptomyces* isolated from seawater sediments, soils and as endophytes associated with plants. Similarly, solvent extracts and culture supernatants from several soil-isolated *Streptomyces* spp. were also active against Coleoptera (Colorado potato beetle, *Leptinotarsa decemlineata*) and Diptera (Mediterranean fruit fly, *Ceratitis capitata*) larvae and adults, respectively [60,61]. One study isolated an endophytic strain belonging to the species *Streptomyces albus*, whose hydrophilic fraction from a solvent extract of the fermentation broth resulted in mortality rates exceeding 80% within 24 h against *Aphis gossypii* (cotton aphid) [62]. Similar mortality rates were observed against green peach aphids (*Myzus persicae*) on bell pepper plants treated with a solvent extract from the fermentation of a *Streptomyces loidensis* strain isolated from agricultural soils [63]. In a notable study, Kim *et al.* [58] screened the filtered ferments of 850 soil-isolated *Actinobacteria* for insecticidal activity against *Plutella xylostella* larvae, identifying 6 strains that exhibited over 80% insect mortality. Amongst these, extract from strain KR0006 (tentatively identified as *Streptomyces cinereoruber*) caused 100% mortality after 3 days of exposure. In another study, Vijayabharathi *et al.* [64] screened 96 *Actinobacteria* strains for bioinsecticide activity against Lepidopteran insects. Fermentation solvent extracts from 15 *Streptomyces* strains exhibited over 50% mortality against second instar *Helicoverpa armigera*, over 38% mortality against third instar *Spodoptera litura* (Tobacco cutworm) and over 50% mortality against 7-day-old *Chilo partellus* larvae (Spotted stalk borer) in artificial diet assays, suggesting that the activity could be attributed to secreted NPs.

Beyond screening ferments or solvent extracts, several studies evaluated the efficacy of *Actinobacteria* spores delivered as a microbial biological control agent. For example, screening a collection of *Actinobacteria* against fruit flies (*Drosophila melanogaster),* Ho *et al.* [65] identified seven strains of *Streptomyces* whose solid-media solvent extracts were toxic when added to the food diet of larval flies. They complemented the study by conducting spore-feeding assays, in which larvae were fed spores added to their artificial diet. The spores also proved toxic, resulting in arrested development and death before reaching adulthood. Spores from one *Actinobacteria* strain (WAC-288) were particularly toxic, causing reduced larval mobility within 3 to 6 h of ingestion and death within 24 h. The authors attribute the insecticidal activity to an NP, described in the ‘Other non-macrolide polyketides’ section. Similar ‘lure and infect’ biocontrol systems were also identified in multiple *Metarhizium* entomopathogen systems [66,67].

In all the studies mentioned above, it was unclear whether the predominant factor for the identification of *Streptomyces* species with insecticidal activity, as opposed to other *Actinobacteria* taxa, was due to an overrepresentation of *Streptomyces* species in collections*,* environmental isolation methods favouring this genus, the use of fermentation methods being more conducive to *Streptomyces* growth or specialized metabolite production or a genuine reflection of a lack of insecticide activity from non-*Streptomyces* species. It is likely that the first applies, as a review of the *Actinobacteria* biopesticide literature (i.e. biofungicides) reported that nearly 40% of bioactivity studies describe *Streptomyces* species, with other individual genera accounting for no more than 10% of remaining studies [20,68].

### Insecticidal natural products from *Actinobacteria*

While microbial biopesticides consist of living cultures, their efficacy is often attributed to the NPs synthesized by the microbe. These NPs act as a pharmacopoeia for modern life science. Natural products from *Actinobacteria* exhibit remarkable chemical diversity, encompassing nearly every class of NP, including peptides, alkaloids, aminoglycosides, polyketides, non-ribosomal peptides and nucleosides, among others. In the context of crop protection and insect pest control, the phylum remains extremely relevant, thanks to multiple discoveries leading to successful insecticidal NPs from *Actinobacteria*. At the forefront of these discoveries are the mectin- and spinosyn-type natural products, which have resulted in a range of natural, semi-synthetic and fully synthetic insecticides [69]. Both mectins and spinosyns are biosynthesized through a polyketide synthase biochemical mechanism, a common biosynthetic pathway in *Actinobacteria*. In fact, many other insecticidal polyketide NPs have been isolated from *Actinobacteria* [49].

Insecticidal bioactive compounds from *Actinobacteria* are not confined to polyketide compounds. The StreptomeDB database [70], a comprehensive resource for *Streptomyces* natural products, lists 95 natural products associated with various pesticidal activities, many of which are not polyketides. Considering that the most well-studied *Streptomyces* genus constitutes only a small fraction of the phylum and much of the biosynthetic diversity within the genus remains underexplored, it is evident that the NP diversity within the *Actinobacteria* phylum is extensive, presenting a promising potential for novel insecticides.

The following subsections present relevant NPs or NP families with insecticidal compounds derived from *Actinobacteria*. They have been organized into chemically similar groups or according to key aspects of their discovery, such as the research history that led to the identification of their insecticidal properties. A non-exhaustive list of NPs sourced from the literature, which have reported insecticidal properties from *Actinobacteria*, is displayed in a table, along with structures of each molecule in File S1 (available in the online Supplementary Material). Natural products with insecticidal, acaricidal or nematicidal bioactivity are considered, as well as bioactivity data from other arthropod experiments, such as brine shrimp or mosquito larvae bioassays. During the compilation of this data, several trends became evident:

- The insecticidal activity of most isolated and structurally elucidated NPs remains unreported, likely because they have never been tested on insects, and/or a tendency in the literature to overlook negative results.
- Modes of action of NPs are rarely reported or investigated in publications, and when they are, they are often extrapolated from other *in vitro* assays. The MoA has been documented here for NPs where information is available.
- The stability or shelf-life of NPs is rarely reported and presents a double-edged sword. For instance, with avermectins, the initial low thermal stability was improved through semisynthetic methods. Conversely, their high sensitivity to UV light is considered advantageous in broadacre crops, as it reduces the risk of persistent environmental effects.
- Increased commercial and scientific interest in a particular NP often coincides with the discovery of more class analogues or congeners. For example, StreptomeDB lists 41 natural products with similar substructures to avermectin and milbemycin, whereas for other insecticides, only 1 congener may be reported. This suggests a significant potential for the existence of new congeners of under-studied NPs that may exhibit superior bioactivity compared to the original discovery.
- For many insecticidal compounds (along with insecticidal spores or ferments discussed in the ‘*Actinobacteria* inoculants or extracts with bioinsecticide activity’ section), the issue of general cytotoxicity presents a significant bottleneck. Numerous publications have not specified whether the compound in question is toxic to other eukaryotes. Others, such as the antimycins, are well-known toxins to various organisms. In any case, off-target cytotoxic effects might be reduced through further development of the lead product, such as via synthetic or semi-synthetic means or via the discovery of other natural congeners, which possess minimal off-target effects.
- Small, volatile terpenes do act as insect attractants or deterrents, such as borneol and geosmin; however, there are few, if any, insecticidal terpenes identified from *Actinobacteria*. This contrasts with the variety of insecticidal terpenes found in plants, such as polygodial and pyrethrins. It remains unclear whether this discrepancy is due to a sampling bias or a genuine lack of bioactive terpenes from this bacterial phylum.

#### The mectins: abamectin, milbemycin, meilingmycin and nemadectin

**Fig. 1.**
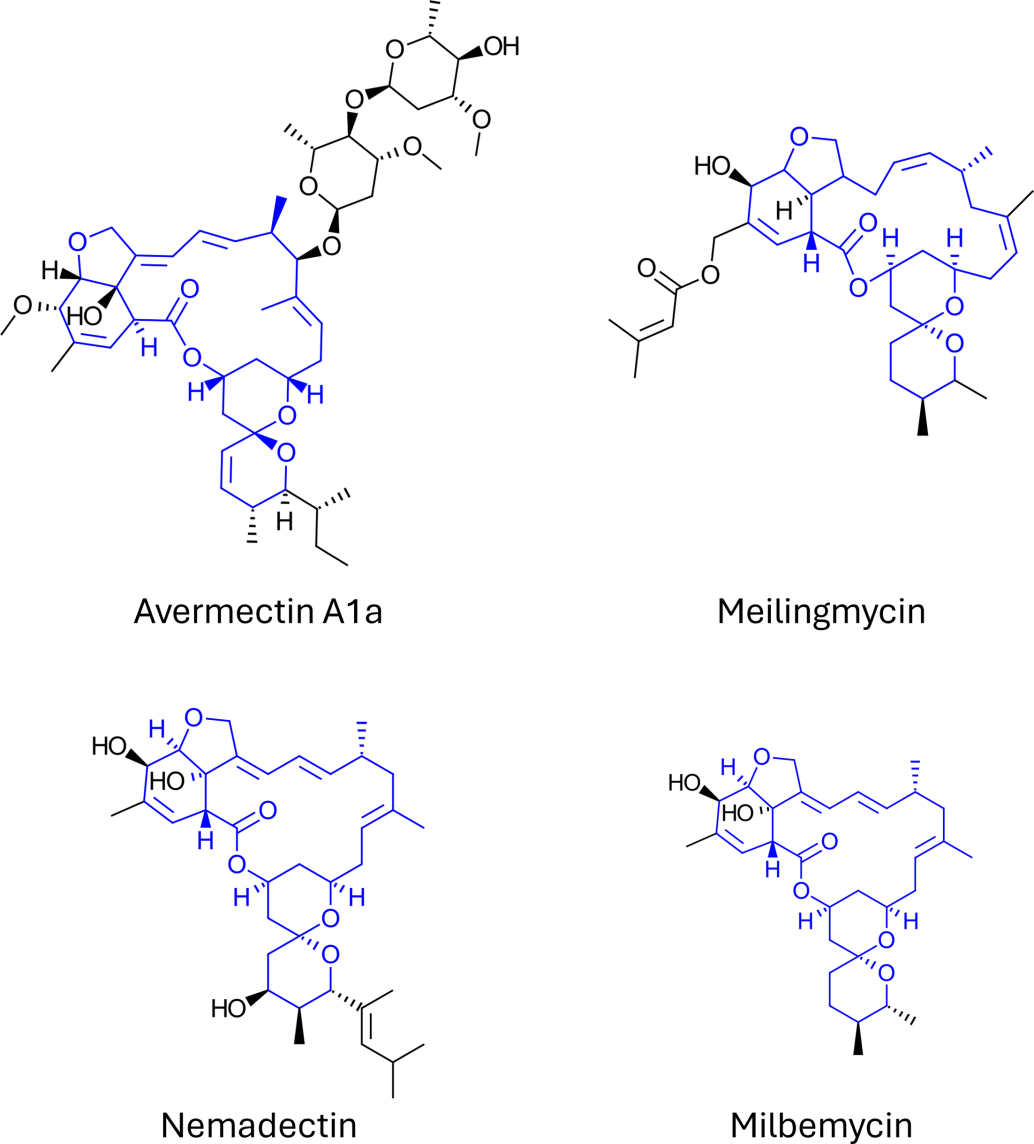
Mectin-type insecticidal natural products. The common carbon skeleton is coloured blue in each molecule.

The mectin family is extensively researched and discussed in detail in other publications [49,51, 71]. Given the volume of literature on the subject, only a succinct summary of the chemical family is provided here. Avermectins are macrocyclic lactones (macrolides), first discovered from a culture of *Streptomyces avermectinius* (ex. *S. avermatilis*). Subsequent discoveries of milbemycin, meilingmycin and several dozen other compounds from *Streptomyces* have built upon this original discovery. All these compounds feature a 16-membered ring with various modifications, cyclizations, glycosylations and side chains (Fig. 1). According to StreptomeDB [70], over 40 NPs with the same carbon skeleton as avermectin have been reported from *Streptomyces* cultures, such as meilingmycin and milbemycin.

Avermectin-like structures exhibit a high affinity for muscular neurons of particular insects and are agonists for GABA-gated chloride channels [8]. This MoA leads to an elimination of signal transduction followed by eventual death. They exhibit activity towards insect pests of the orders Lepidoptera, Isoptera, Diptera, Hymenoptera, Coleoptera and Blattodea [49,72]. The mectins also exhibit broad-spectrum antiparasitic and anthelmintic activity, revolutionizing the veterinary world with the ability to treat both external and internal parasites like worms, ticks and flies [73]. The impact of mectin natural products on health and medicine led to Campbell and Ōmura receiving the Nobel prize for their contributions to the discovery [74].

Abamectin was the first commercial product based on avermectins, comprising 80% avermectin B_1_a and 20% avermectin B_1_b, and is used as a biocontrol agent for insects and phytophagous mites [47,71]. Most modern crop protection products are semi-synthetic mectins, offering improved thermal stability and bioactivity compared to their natural precursors [69].

Although abamectin is reported to be toxic to pollinators through contact or ingestion, it degrades when exposed to UV light, undergoing rapid photodegradation within 24–28 h after application [75]. For this reason, this sensitivity is environmentally advantageous in broadacre crop systems.

#### Spinosyn

The discovery of spinosyn NPs, another highly modified macrolide-type molecule, highlights the potential of successful collaboration between industry and academia [76]. Spinosyn will only be briefly mentioned here; however, much more information is publicly available [49,69, 76, 77]. It was originally discovered from a culture of the rare Actinobacterium *Saccharopolyspora spinosa*, underscoring the biochemical potential of *Actinobacteria* beyond the well-studied *Streptomyces*. According to Kirst [76], early bioassays showed only modest bioactivity due to minute quantities of extremely potent spinosyn in *S. spinosa* ferments. This mild bioactivity was not overlooked, and the persistence of the researchers led to the successful discovery of spinosyn derivatives now used to control a wide range of insect and arachnid pests.

**Fig. 2.**
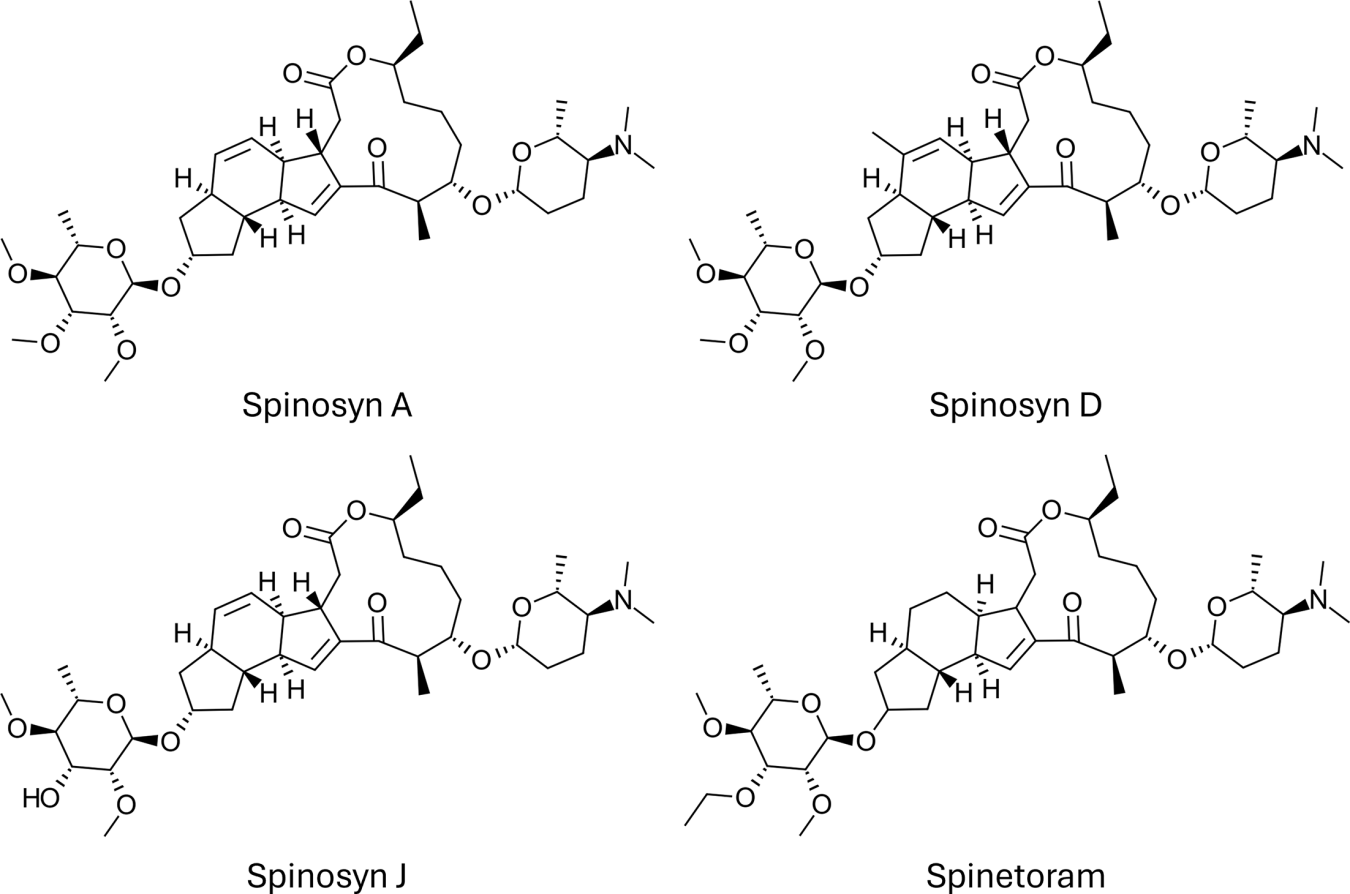
Variants of the spinosyn family. Spinosyn A and spinosyn D constitute a mixture referred to as spinosad, typically in a 4:1 spinosyn A/D ratio. The semi-synthetic product spinetoram is derived from the natural product spinosyn J.

Several dozen natural spinosyn congeners have been discovered, with spinosyn A and D (the mixture known as spinosad, Fig. 2) being the most common commercially available. Many thousands of semi-synthetic derivatives of spinosyn have been developed as part of structure-activity relationship studies [69], with some being commercialized, such as spinetoram (an alkylated and hydrogenated product chiefly derived from spinosyn J, Fig. 2).

Spinosyns act on the nicotinic acetylcholine receptor of insects, leading to neuronal excitation. This MoA is different from other nicotinic receptor insecticides, acting at a different site. There is also evidence that spinosyns act on GABA receptors [50]. Resistance has been detected in pest populations, and in some cases, a genetic basis for resistance has been identified [50]. Spinosyns can function as both contact and ingestion-based bioinsecticides [38].

#### Other polyketide macrolides

Beyond spinosyns and mectins, there are numerous other insecticidal macrolides reported from *Actinobacteria*. Their biological properties are diverse, including insecticidal effects, but they also raise concerns about off-target effects such as antibacterial, antifungal or generally cytotoxicity. Nevertheless, this diversity of insecticidal compounds is still being explored and represents unique scaffolds from which novel insecticides may be developed. In some cases, a biological product with both antifungal and insecticidal properties could be particularly beneficial, such as for controlling wood-boring beetles. These beetles damage trees through primary chewing, which is then infected by the symbiotic fungi they carry. Serious pests such as the polyphagous shothole borer (*Euwallacea fornicates*) and bark beetles (e.g. *Ips grandicollis*) kill trees in this manner and could be key target species for any dual-purpose biocontrol agent.

One example is aculeximycin, a 26-membered macrolide with numerous glycosylations. First isolated in 1,983 from a species of *Kutzneria* [78], its corresponding biosynthetic gene cluster was identified in 2014 [79]. The compound showed very high toxicity to mosquito larvae (0.66 p.p.m.) and also exhibited antibacterial activity (against *Staphylococcus* and *Bacillus*) and antifungal activity (against *Candida* and *Saccharomyces*) [78].

#### Polyether ionophores

**Fig. 3.**
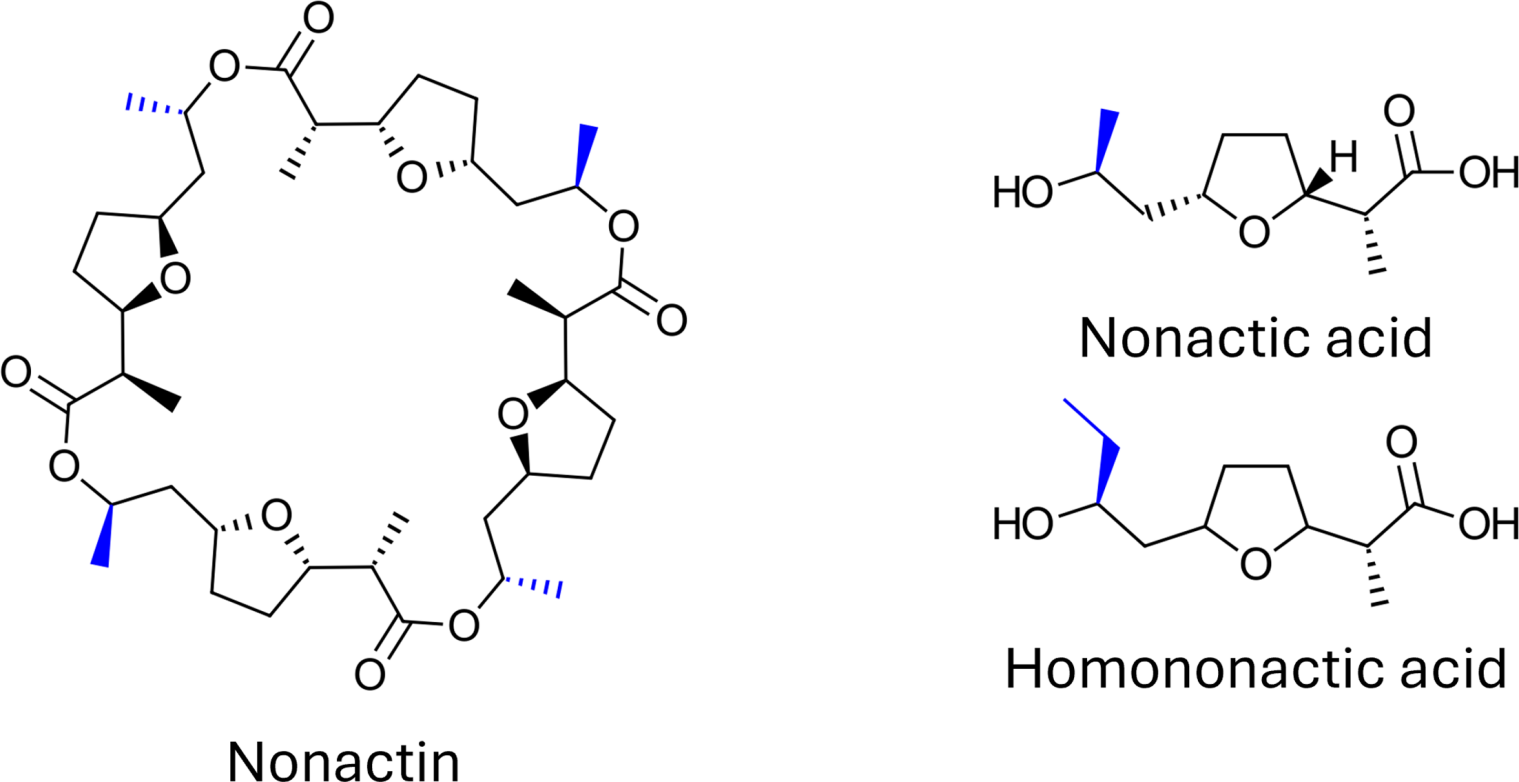
Nonactin (left), a polyether ionophore natural product with insecticidal activity. Nonactin and respective congeners are biosynthesized from varying ratios of nonactic and homononactic acid subunits (right). The functional group variation (methyl or ethyl side chains) resulting from subunit use is shown in blue. Nonactin and its congeners also produce these subunits during decomposition.

Amongst the polyether class of polyketides, the polynactin family is notable for its insecticidal activity. The family consists of five compounds – monactin, dinactin, trinactin, tetranactin and nonactin – which differ in the number of ethyl groups instead of methyl groups, resulting from the ratio of nonactic acid and homononactic utilized during biosynthesis (Fig. 3). Polynactins are already registered as a crop protection product to control mites [49]. They are also active against Coleoptera, Lepidoptera and Hemiptera, including five aphid species [63]. Several related polyethers have shown biological activity in brine shrimp assays [80].

In 1992, Jizba *et al.* demonstrated that the nonactic and homononactic acid subunits of polynactins are also insecticidal [81], with bioactivities comparable to the synthetic insecticide metathion but with lower antibiotic activity. They also noted that biological degradation of polyether antibiotics produces nonactic and/or homononactic acid monomers, depending on the polyether in question. From a biocontrol perspective, this family of polyethers presents an opportunity where a bacterial ferment could yield an array of bioactive NPs that remain insecticidal even as they degrade. These polyether NPs are considered as ionophores (along with other polyethers such as indamycin and dianemycin; see Table 2), acting on mitochondrial membranes of eukaryotes to inhibit growth. This represents a novel MoA for insect control, although it remains hypothetical and has not been corroborated *in vivo*.

**Table 2.** List of natural products with documented insecticidal activity from *Actinobacteria* Natural products are grouped by their respective chemical classes, with insect orders known to be susceptible to each NP listed as comprehensively as possible. Only a single listing is provided for those NPs that have multiple congeners or isomers with insecticidal activity (such as avermectin).

Compound	Reference	InChI key	Susceptible arthropod order (or more specific taxa)
**Polyketide macrolides**			
5′-Epi-SPA-6952A	[126]	BATWHDBFBKQYSY-KAKKGFKTSA-N	Lepidoptera
Aculeximycin	[78]	VJKZKLDZOAFAEE-HVJLGTRBSA-N	Diptera (Culex)
Avermectins	[69]	AFSHKCWTGFDXJR-HLFFGZBOSA-N	Blattodea; Coleoptera; Diptera; Hymenoptera; Isoptera; Lepidoptera; Nematoda
Bafilomycin	[127]	KFUFLYSBMNNJTF-ANDWMEETSA-N	Nematoda
Faeriefungin	[128]	GBVIQYQFMPWELT-VNYSJGGHSA-N	Diptera (Aedes); Nematoda;
Fungichromin	[129]	AGJUUQSLGVCRQA-SWOUQTJZSA-N	Nematoda
Leucanicidin	[130]	KFLYTTUTONVURR-RZZUOCMJSA-N	Lepidoptera; Nematoda
Meilingmycin	[131]	QSHJCXWGTCXGAX-ZCVXJSSDSA-N	Acari; Hemiptera (Aphis); Lepidoptera
Milbemycin	[132]	ZLBGSRMUSVULIE-GSMJGMFJSA-N	Acari; Hemiptera (Aphis); Lepidoptera
Nemadectin	[133]	YNFMRVVYUVPIAN-AQUURSMBSA-N	Acari; Nematoda
Ossamycin	[134]	XGECDDPXIKFBTE-ZVNFYDRVSA-N	Diptera
Seco-nemadectin	[133]	WDWFRIMNWVDXGF-YQERLSOISA-N	Acari; Nematoda
Spinosyns	[77]	SRJQTHAZUNRMPR-UYQKXTDMSA-N	Diptera; Hymenoptera; Lepidoptera; Siphonaptera; Thysanoptera
Tartrolone C	[106]	WOJDVSNFPBXUGQ-TVWAQYCBSA-N	Lepidoptera
**Polyether polyketides**			
Dianemycin/nanchangmycin	[135]	XMAIRYYXDCNFKP-SEDNIUBGSA-M	Hemiptera; Diptera
Indanomycin	[136]	BAIPOTOKPGDCHA-DFPVWRGBSA-N	Lepidoptera
Nonactin (and polynactins)	[137]	RMIXHJPMNBXMBU-QIIXEHPYSA-N	Acari; Hemiptera (Aphis)
Nonactic acid	[81]	IVOODSRSVJPWLY-UYXSQOIJSA-N	Coleoptera; Hemiptera (Aphis); Lepidoptera
Homononactic acid	[138]	HTCUURQJNZBKIA-XHHQTKHESA-N	
Other nonactic/homononactic acid-derived ethers	[80]	n/a	Arthropoda
**Other polyketides**			
Aureothin	[139]	GQKXCBCSVYJUMI-WACKOAQBSA-N	Nematoda
Cosmomycin D	[65]	DKWBRHNUUTWKAG-JNZMSYBZSA-N	Diptera
Manumycin	[140]	TWWQHCKLTXDWBD-MVTGTTCWSA-N	Coleoptera; Lepidoptera
Piericidin	[141]	BBLGCDSLCDDALX-LKGBESRRSA-N	Acari; Blattodea; Diptera; Hemiptera (Aphis); Lepidoptera
Spectinabilin	[142]	IZICQJAGBLBAMJ-QDYRYYKCSA-N	Nematoda
Strekingmycin	[82]	n/a	Hemiptera (Myzus)
**Peptides (including Non-ribosomal peptides**)			
Diketopiperazines	[101]	CUVKAUWOMPJEMI-ROUUACIJSA-N	Lepidoptera (Mythimna); Nematoda
Microviridin	[93]	GZWAMQUNOCFCLH-IPFDCVSDSA-N	Arthropoda (Daphnia)
Orfamide	[94]	AFOLBAYDTRBYBA-NZZMYBQCSA-N	Lepidoptera
Respirantin	[95]	HJNZTHHOZWMVPB-WCSXKIRISA-N	Lepidoptera (Mythimna)
SLP1-H008	[92]	n/a	Hemiptera (Lipaphis)
Quinomycin A	[100]	AUJXLBOHYWTPFV-UHFFFAOYSA-N	Diptera (Culex); Hemiptera (Aphis); Lepidoptera
**Glycosides**			
Gualamycin	[143]	IZZRXUHORVVNQL-LLEMLHBASA-N	Acari
Racemomycin /Nourseothricin streptothricin	[87]	NRAUADCLPJTGSF-WJPMJIHPSA-N	Blattodea; Diptera
Validamycin	[85]	JARYYMUOCXVXNK-CSLFJTBJSA-N	Diptera (Aedes); Hemiptera (Diaphorina)
**Others (including hybrids**)			
Altemicidin	[144]	VZRFZUPFQKSXPV-VPFIQFBESA-N	Acari, Arthropoda
(2S,5R,6R)−2-hydroxy-3,5,6-trimethyloctan-4-one	[145]	HHSDJVQAEHOWEL-VYQDEHIVSA-N	Acari; Diptera (Culex)
5-(2,4-dimethylbenzyl) pyrrolidin-2-one (DMBPO)	[146]	PUUWMFZWQUAGCG-UHFFFAOYSA-N	Acari; Diptera (Culex)
10-(2,2-dimethyl-cyclohexyl)−6,9-dihydroxy-4,9-dimethyl-dec-2-enoic acid methyl ester	[147]	n/a	Nematoda
Antimycins	[91]	NAEDADOYRYAJDM-UHFFFAOYSA-N	Acari; Diptera; Lepidoptera; Thysanoptera
Fervenulin	[103]	RRTKVYSLIGQWCO-UHFFFAOYSA-N	Nematoda
Gougerotin	[148]	AMNAZJFEONUVTD-QJHHURCWSA-N	Hemiptera (Myzus), Lepidoptera
Jietacin	[105]	BKQGCLAUQLABKR-UHFFFAOYSA-N	Nematoda
*N*-(1S-((4R, 5R)-2,2-dimethyl-5-undecyl-1,3-dioxolan-4-yl)−2-hydroxyethyl) stearamide	[149]	YSGBEFDGBKQJTK-VUHKNJSWSA-N	Lepidoptera
Nikkomycins and polyoxins	[90,150]	WWJFFVUVFNBJTN-VHDFTHOZSA-N	Acari, Coleoptera, Lepidoptera
O-(2-(3-methyloxiranyl) cinnamoyl) threonine	[151]	ODWSYVQLIHQQPN-AIUXAJILSA-N	Acari
Sinefungin	[108]	LMXOHSDXUQEUSF-YECHIGJVSA-N	Lepidoptera; Orthoptera
Teleocidin	[104]	PEYTUVXFLCCGCC-YGHSORLUSA-N	Nematoda
Valinomycin	[97]	FCFNRCROJUBPLU-UHFFFAOYSA-N	Acari; Coleoptera; Diptera (*Aedes*)

#### Other non-macrolide polyketides

**Fig. 4.**
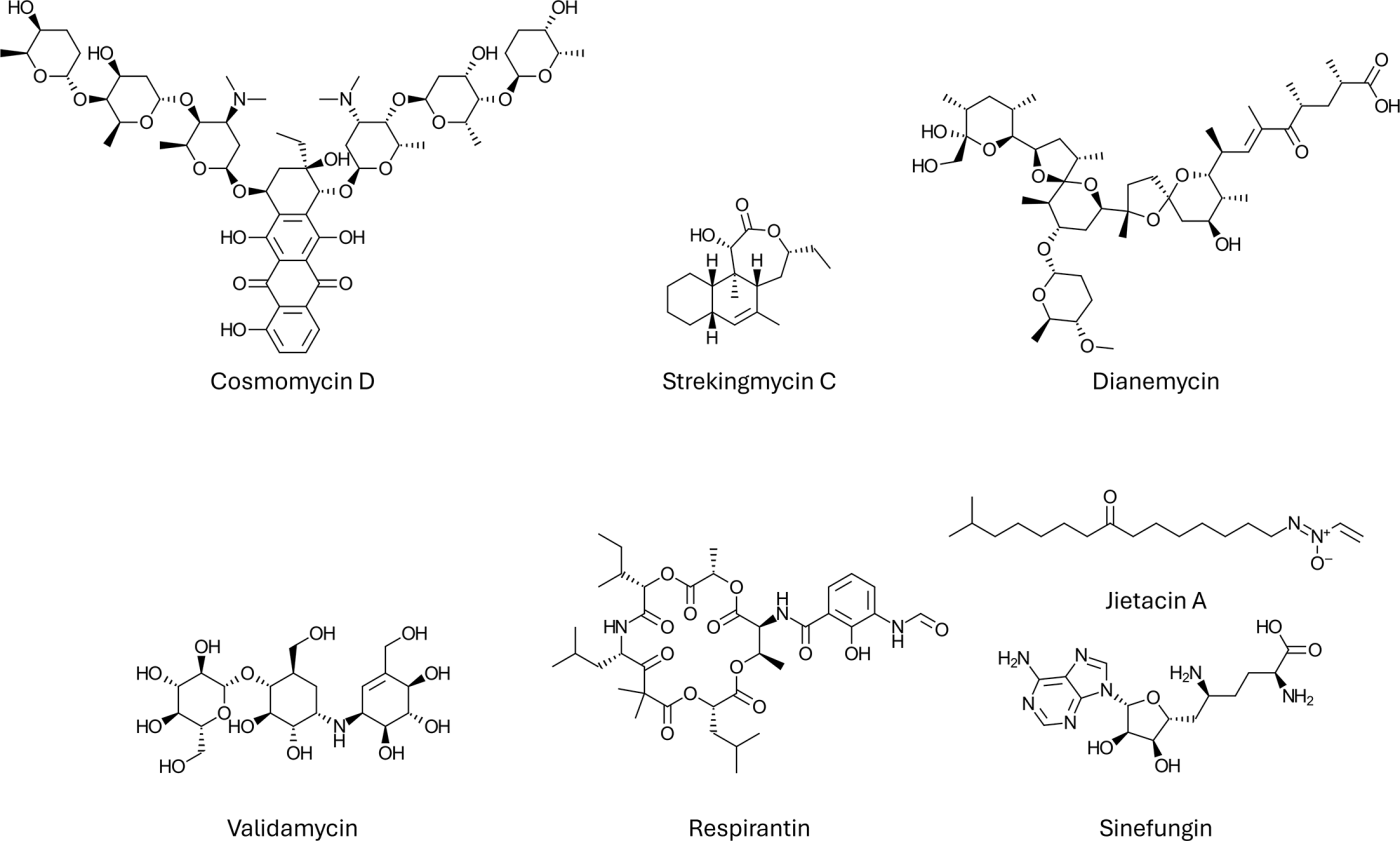
Representative insecticidal natural products from *Actinobacteria*, illustrating the structural diversity of bioactive molecules. Insecticidal products stem from a wide range of biosynthetic classes, including polyketides, such as cosmomycin D and strekingmycin C; polyethers such as dianemycin, aminoglycosides (validamycin), non-ribosomal peptides (valinomycin), nucleosides (sinefungin); and others, such as jietacin A.

Several insecticidal non-macrolide polyketides have also been identified. In 2020, Ho *et al.* [65] conducted a study screening of 56 *Streptomyces* species for insecticidal activity against 6 different *Drosophila* species. They identified a highly potent strain, with its bioactivity attributed to the polyketide cosmomycin D (shown in Fig. 4). Experiments showed that fruit flies preferred a diet containing these spores over uninoculated foods, an effect mediated by the volatile compound 2-methylisoborneol, common to most *Actinobacteria*. After spore consumption, 100% mortality was rapidly achieved. This study not only identified a new insecticidal NP but also demonstrated the potential of spore-based bioinsecticides.

Another interesting polyketide, strekingmycin (Fig. 4), was recently reported by Wang *et al.* [82]. This NP was purified from a *Streptomyces* strain originally isolated from an earthworm. Eight strekingmycin congeners have now been described [82,83]. In 2023, Wang showed strekingmycin C had the best activity against the Green peach aphid, *M. persicae,* while work from Liu *et al.* showed that these polyketides were also toxic to the Greenhouse whitefly, *Trialeurodes vaporariorum*.

#### Repurposed antibiotics

Many reports highlight the insecticidal activity of compounds previously researched as antibacterial or antifungal compounds. While this raises concerns about off-target effects, it also suggests untapped novel modes of action and indicates that some NPs have yet to be tested against insects. This approach, known as cross-indication testing, has led to several successes with synthetic molecules and is a promising discovery route for NPs [84].

The aminoglycoside antibiotic validamycin (Fig. 4) is one example of antibiotic repurposing. It is used in agriculture to control fungal diseases such as sheath blight by inhibiting the enzyme trehalase, which digests trehalose sugars when fungi infect a host plant. Trehalase activity is also common in insects, and its inhibition via validamycin has been proposed as a novel insect control method, such as for the sap-sucker *Diaphorina citri* [85]. Similarly, antimycin and streptothricin-type antibiotics (such as racemomycin), both isolated from *Streptomyces*, have shown insecticidal properties [86,87]. This research is mirrored by chitin synthase inhibitors due to the chitin synthase requirements shared by fungi and insects. For example, the antifungal nikkomycin appears to have insecticidal properties [88,89], along with the structurally related polyoxins [90], both derived from *Streptomyces*. Interestingly, different polyoxin congeners showed striking variation in insecticidal activity, showing that specificity can be achieved with chemical diversity [88]

Variants of antimycins offer variability of bioactivity [86]. Kim *et al.* [91] isolated five antimycins from the solvent extracts of *Streptomyces* sp. AN120537 ferments. These antimycins exhibited insect juvenile hormone antagonist and potent insecticidal activities against *Aedes albopictus* (mosquito). Detailed studies on agricultural insect pests revealed differential toxicity, with over 50% pest mortality recorded for all five antimycins against *Plutella xylostella* (Diamondback moth); A_3_, A_4_ and A_5_ against *Frankliniella occidentalis* (Western flower thrip); and A_2_ and A_3_ against *Tetranychus urticae* (spider mite).

#### Peptides

Compared to polyketides, insecticidal peptides of actinobacterial origin are significantly underutilized. Their potential lies in the chemical diversity offered by permutations of peptide sequences and post-translational modifications, such as cyclization and tailoring enzymes. Despite this potential, few insecticidal peptide NPs are reported from *Actinobacteria*. Only one ribosomally synthesized peptide from *Actinobacteria* was identified through our literature surveys: a 15-amino acid peptide with activity against the mustard aphid *Lipaphis erysimi*. Although it had modest bioactivity, it showed good thermal and pH tolerance [92]. From other phyla, promising peptides include microviridin from *Cyanobacteria* [93] and orfamides from *Pseudomonas* [94].

Non-ribosomal peptides (NRP) are synthesized using amino acid subunits and biosynthetic enzymes, unlike ribosomes that synthesize canonical peptides and proteins. Non-ribosomal peptides can be heavily modified and incorporate other biosynthetic features, and the molecule’s backbone can contain bonds other than peptide bonds. This is evident in the structures discussed here, which contain ester bonds, leading to their classification as depsipeptides. Several insecticidal NRPs have been isolated from *Actinobacteria*, including the cyclic NRP depsipeptide respirantin (Fig. 4). This small cyclic peptide was discovered during screening for bioactivity against the Lepidopteran *Mythimna seperata*, resulting in 100% mortality at a dose of 500 p.p.m. in artificial diets [95]. It proved to be very thermostable, even after autoclaving. The structure of respirantin is analogous to fungal toxins like bassianolide, beauvericin and enniatin [96], which have insecticidal activity, as well as the hybrid NRP-polyketide antimycins. However, unlike enniatin and antimycin, respirantin appears to have relatively mild off-target effects.

Valinomycin, another large cyclic NRP with broad bioactivity, including impressive bioactivity against a variety of insects, has been isolated from a range of *Streptomyces* spp*.* [97,98]. Interestingly, some *Streptomyces* strains produce both valinomycin and bafilomycin, highlighting the ‘multidrug’ potential of biocontrol agents [99]. In addition, the more complex NRP quinomycin, isolated from *Streptomyces* strains, was found to have insecticidal activity against Lepidoptera and aphids [100].

Cyclic dipeptides, or diketopiperazines, consist of two peptides cyclized to produce a diketopiperazine heterocycle. Despite their simple backbone, they exhibit diverse bioactivities, including phytotoxic, antibacterial, cytotoxic and insecticidal effects [101]. For example, cyclic tryptophan–phenylalanine showed antifeedant activity against *Helicoverpa armigera* [102].

#### Other natural products

Several other bioactive NPs are worth mentioning for their unusual chemistry. For example, Ruanpanun *et al.* [103] reported the nucleic acid-derived fervenulin, isolated from a *Streptomyces* isolate with activity against plant parasitic nematodes. Also with activity against nematodes, Kang *et al.* [104] identified an indole terpene, teleocidin, from a *Streptomyces* isolate.

Sugawara *et al.* [105] isolated four variants of the NP jietacin (jietacin A is shown in Fig. 4). These compounds feature a long alkyl chain with minor modifications attached to the highly unusual vinylazoxy functional group. In bioassays against the Pine wood nematode (*Bursaphelenchus lignicolus*), the jietacins demonstrated ten times greater efficacy than avermectin controls.

In 2003, Lewer *et al.* described tartrolone C, which was derived from a *Streptomyces* culture originally isolated from soil [106]. This dimeric macrolide (also known as macrodienolide) is one of the very few NPs containing a boron atom and displays unique bioactivities, particularly against Lepidopterans.

Sinefungin (shown in Fig. 4), a nucleoside NP first isolated in the early 1970s [107], was later found to inhibit the development of the locust *Locusta migratoria* [108]. *In vitro* experiments indicated that this inhibition was due to the disruption of juvenile hormone biosynthesis. Subsequent studies in the Lepidopteran *Manduca sexta* supported this MoA, with sinefungin treatments resulting in 100% mortality of the moth larvae before pupation [109]. These studies outline a rare case of NP discovery that includes elucidation of the MoA. Juvenile hormone disruptive activity has been observed in several strains of *Actinobacteria* [110], suggesting that this MoA may be relatively common.

## Opportunities for bioinsecticide discovery and adoption

### New sources of *Actinobacteria* bioinsecticides

**Fig. 5.**
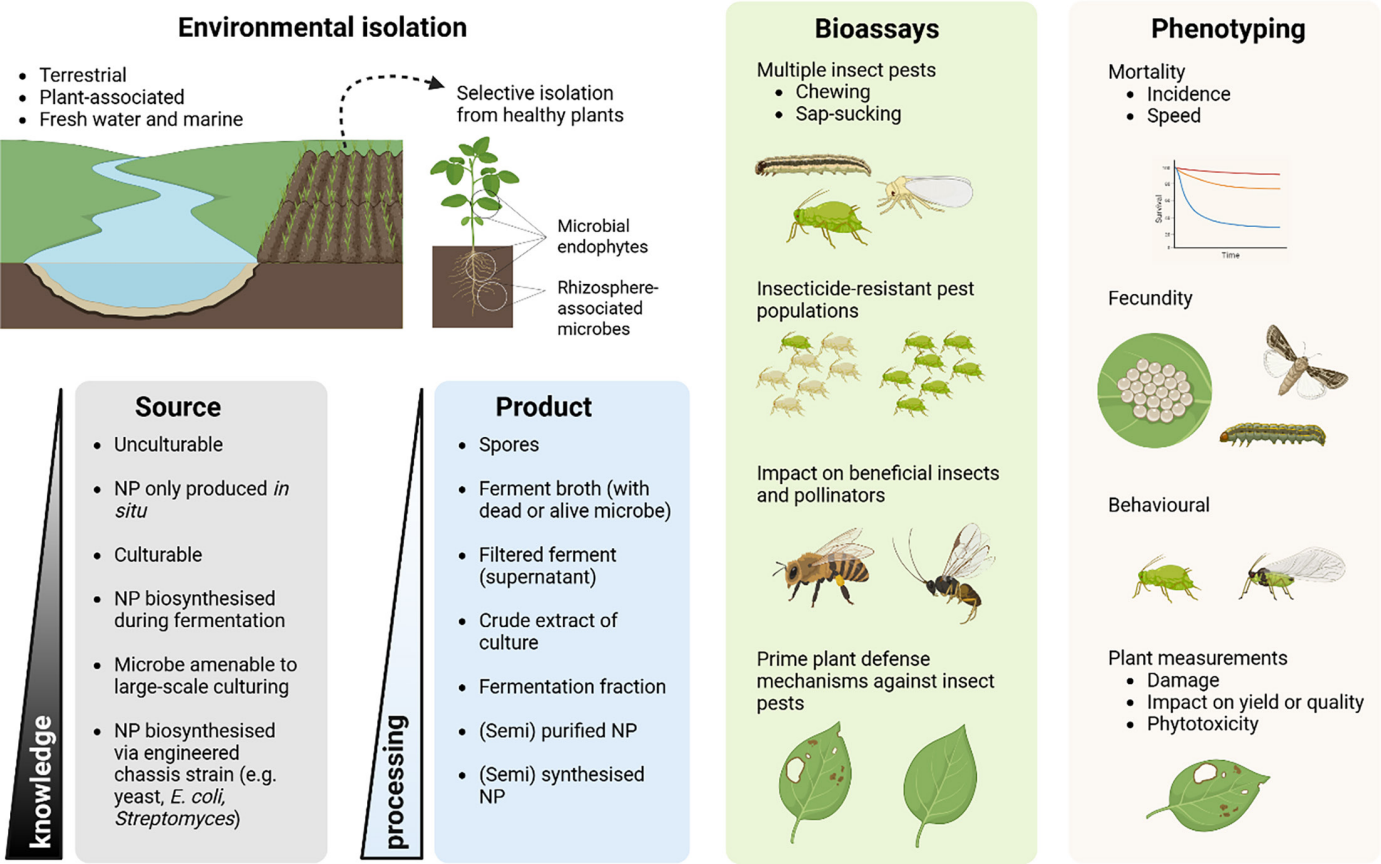
Summary of opportunities for the discovery and adoption of *Actinobacteria*-based or derived bioinsecticides. The integration of high-throughput bioassays to assess the effects on multiple insect pests, the diversity of insect populations, non-target organisms and plant priming responses, along with new phenotyping systems, could facilitate the discovery of novel microbial MoA while minimizing impacts on biodiversity. Created in BioRender. Dow, L. (2026) https://BioRender.com/eq1txia*.*

*Actinobacteria* isolated from non-routine sampling sites, such as the plant microbiome, represent a relatively untapped source of potential new insecticidal NPs [45,111] (Fig. 5). There are significant opportunities to explore these niches within plants, where plant endophytes residing in plant cells across various tissues have been less investigated for their metabolic potential compared to culturable microbes from soils or waters [68]. It is hypothesized that such host-associated microbes possess a larger and more diverse range of natural products than free-living microbes [68,112, 113]. These NPs serve various functions beyond general resource competition and microbial defence, focusing on host interactions for survival in both soil and plant environments. If an insecticidal NP is produced within the plant environment at sufficient levels under agricultural conditions, it could serve as an ideal microbial inoculant. One potential method of delivery would be microbial-coated seed treatment for early stages of plant protection or applying the inoculant as a spray at later stages of crop development for ongoing protection. Additional benefits may arise from the microbes’ ability to activate plant defence responses against insect pest attacks [114]. There are also opportunities to characterize new strains of known microbial biopesticides for qualities benefiting commercialization, such as faster growth, higher NP titre or a new biochemistry profile and intellectual property differentiation [115].

A vast opportunity for discovering microbial NPs also exists within the unculturable microbes – those that cannot be isolated from the environment or cultured under routine laboratory conditions. This represents a significant ‘black box’ of unknown NP diversity and products that may only be expressed within specific environmental contexts. It is known that the effects of applying single-strain microbial inoculants to plants in the field do not always reflect laboratory observations [19], leading to reports of variable field efficacy and challenges for on-farm adoption [116]. However, this also suggests that the benefits from microbes depend on their natural environments, including interactions within native microbial communities.

Accessing this ‘hidden diversity’ using advanced omics tools to capture nucleic acid (DNA, RNA) or metabolomics data *in situ* could help discover new bioinsecticides or their respective biosynthetic gene clusters. It would also provide insights into how microbial biopesticides function *in planta* and the environmental conditions that ensure consistent efficacy. This information can be used to improve the application of *Actinobacteria* or other microbes in the field, such as determining the best timing and frequency for application. A meta-analysis of 160 studies on *Streptomyces* strains for fungal control under various growth conditions showed that while *Streptomyces* are effective microbial biopesticides, their success depends on environmental factors, levels of disease pressure, the type of disease and the method of application [117].

### Coupling advances in bioactivity screening

Research often focuses on a single insect pest species, and often just one phenotype, likely due to cost or complexities involved in working with insects. Expanding bioassay data to include multiple insect species, including beneficial species, and investigating phenotypes beyond traditional toxicity or mortality assessments – such as fecundity (the ability to produce offspring) and behavioural changes like the inability to settle, feed or move to alternate hosts – offers significant potential for discovering new MoAs.

To address challenges in screening, bioassays can be miniaturized to reduce cost and incorporate automation in methods, data acquisition and analysis. For instance, image detection tools and machine learning can be utilized to adapt to specific data classifications (phenotypes) for evaluating changes in insect mortality and behaviour. Numerous studies have reported the ability of *Actinobacteria* ferments or their derived extracts to activate plant defence or immunity responses [19,118]. However, few have used this beneficial agricultural trait as a tool to screen strain collections and identify leads that could activate systemic acquired resistance and provide protection against insect pests. To our knowledge, two studies have utilized plant defence bioreporters incorporated within a plant host to detect a microbial biological control agent or its NPs. These studies focused on *Streptomyces* species that enhance plant defence responses against fungal infections [119,120].

In another interesting study demonstrating the high-throughput capabilities of bioreporter systems, Kim *et al.* [91] screened 363 *Actinobacteria* solvent extracts from mycelia against a yeast reporter line, which quantified potential juvenile hormone (JH)-based insect growth regulator activity. The authors identified eight extracts with high levels of JH antagonist activity, two of which showed over 80% mortality against *Plutella xylostella* (diamondback moth).

Finally, cross-indication testing – assessing final compounds alongside their intermediates or by-products from one agrochemical indication against target species of other product lines – is a proven strategy for sourcing lead compounds. This approach has resulted in several important crop protection products, including insecticides derived from herbicide and fungicide chemistry [84].

### Transition from lab to field

Following laboratory or glasshouse-based bioassays, candidate bioinsecticides should be validated under field conditions through the development of field protocols that reflect their MoAs. Too often, bioinsecticides and biopesticides more generally are tested stand-alone and rated against chemical insecticides using field protocols developed for chemical evaluations [115]. In these scenarios, efficacy endpoints typically reflect those for chemical insecticides, such as pest damage or pest numbers, and overlook sublethal effects or behavioural or reproductive impacts. These also overlook longer term farming systems benefits from bioinsecticide incorporation into IPM plans, such as resistance management, less impact on pollinator services and beneficial insects, and improved farm biodiversity.

Timing, dose and application method are critical to success. For example, if the application does not coincide with the most suitable pest life stage for the bioinsecticide MoA or if the application does not account for a microbial bioinsecticide potentially being slower to kill, pests may not be appropriately controlled. This research is best conducted with grower input, so the protocols developed are practical and address grower needs. Once a bioinsecticide is commercialized, field trials run in conjunction with growers should continue as a feedback loop with industry to increase grower awareness, education and knowledge on how and when to use a bioinsecticide.

### Supporting adoption and sustainable crop production

We highlight in previous sections large bodies of research that have led to the successful development and commercialization of *Actinobacteria*-derived bioinsecticides, while other areas of discovery have stalled and not been adopted. While reasoning for much of the latter is often not publicized, bioinsecticide leads have likely failed to meet field efficacy, storage or stability requirements. Sometimes this is a reality, or a wrongful expectation. Improvements in formulations can help address the former, which, when coupled with tailored field protocols, can improve efficacy and reduce inconsistency [6,7]. Broadening bioassays against different arthropod species, evaluating bioinsecticides against insecticide-resistant pests or quantifying reduced impact on beneficial insects or pollinators can be used as metrics to differentiate bioinsecticides from traditional chemical controls.

The adoption of *Actinobacteria* biopesticides as microbial inoculants or NPs offers opportunities for insect control where current or future conventional chemical control options are unavailable or unsuitable. This may be due to regulations, resistance development, toxicity to non-target organisms, residue limits or application timings where chemicals are not permitted. Many microbial-derived NPs leave little to no toxic residues and decompose quickly, resulting in zero or low re-entry and pre-harvest intervals. For example, abamectin is stable in storage at ambient conditions for up to 2 years after manufacture but degrades relatively quickly on plant surfaces due to photodegradation [69]. It also breaks down in soil and water. While rapid photodegradation can pose challenges for the commercialization of bioinsecticides, it contrasts with many traditional chemical controls, offering reduced environmental persistence and better residue management. This contributes to improved soil health, water quality and ecosystem biodiversity while effectively controlling insect pests.

The success of avermectins and spinosyns has demonstrated the potential of *Actinobacteria*-derived NPs as environmentally safe, biodegradable and target-specific pesticides [121]. Their unique modes of action may help reduce the likelihood of insects developing resistance and support sustainable agriculture by decreasing reliance on synthetic chemical pesticides [122,123]. However, as with all controls, care must be taken not to overuse a single active ingredient. For example, amongst options for organic growers, spinosad is one of the most effective bioinsecticides for controlling thrips, coleopteran and lepidopteran pests. Its extensive use in potato fields has led to resistance development in some populations of Colorado potato beetle in the USA [122,123]. Although such instances of resistance are rare, with few examples reported for commercialized biopesticides, they underscore the need for proactive resistance management and stewardship, including developing frameworks to maintain the durability and longevity of these products [33]. This also highlights the advantage of developing bioinsecticide mixtures from fermentation extracts or microbial inoculants, which can reduce the potential for resistance development in insect pest populations through multiple modes of action.

Opportunities also exist for combination products with other bioinsecticides or chemical insecticides in integrated pest management programmes, thereby reducing chemical loads and mitigating resistance development. This approach has been readily adopted across some broadacre crops, for instance, corn, soybean and cotton, where microbial biopesticide seed coatings to protect crops at planting time from destructive insects, nematodes or root diseases are stacked with chemical pesticides on the seed [115].

## Conclusions

A primary aim of this review was to present an overview of the bioactivity data associated with Actinobacterial bioinsecticide products or the ferments and NPs derived from them. For NPs, we identified compound classes and structural variants with significant insecticidal activity, highlighting knowledge gaps and promising areas for further research. We found that the majority of studies reported activity from fermentation extracts or from semi-purified specialized metabolites, rather than assessing the impacts of Actinobacterial inoculants (live cells) on pests. This is in stark contrast to other bioinsecticide studies where microbial biopesticides based on other bacteria (e.g. *Bt* strains) or entomopathogenic fungi dominate. This trend reflects the focus of Actinobacterial research on the phylum’s broad NP diversity and isolation of new NPs, similar to clinical research. Thus, the study of Actinobacterial inoculant solutions (e.g. spores) represents an underexplored opportunity for bioinsecticide discovery, alongside the co-development of in-field evaluation protocols suited to microbial MoAs. From the screening of Actinobacterial collections, 1–12% of strains exhibited bioinsecticide activity, with studies reporting an average 9% hit rate. Due to the diversity and high number of individual metabolites produced by a single isolate, these hits could simply be producing NPs toxic to many organisms (including crops), and to rule this out, detailed studies of prospective bioinsecticides are required. This potential downside of spore-based products is balanced by synergistic effects, as shown by Ho *et al.* [65]. Most ctinobacterial bioinsecticide studies focused on strains isolated from soils and often described *Streptomyces* species, highlighting opportunities for discovery amongst other less studied genera such as *Micromonospora* or *Microbispora*, or more unusual genera such as *Kutzneria* or *Saccharopolyspora*, as was the case for the discovery of spinosyn.

Our review revealed a severe lack of studies exploring peptide NPs as potential bioinsecticides. Most studies targeted pests of the Lepidoptera and Diptera orders, with fewer studies (~50% less) assaying Hemiptera or Coleoptera insects. Agricultural pests of the Nematoda order were commonly assayed, followed by Arachnida. Combined, these findings highlight a gap in screening actinobacterial bioinsecticides against important agricultural insect pests such as aphids, whiteflies, leafhoppers, weevils and beetles.

It is evident from the literature and the ongoing generation of omics data in the public domain that the NP diversity of *Actinobacteria* is extensive, and the potential for novel bioinsecticides is very high. There is also significant potential for the discovery of improved bioactivity or other important agricultural traits (e.g. stability) in new congeners from understudied NPs. An ongoing challenge or roadblock to discovery or improvement remains the limited range of insect pests screened or reported. Without this information, many potential bioinsecticide NPs may be overlooked for future development. This includes a lack of studies exploring *Actinobacteria* or their NPs for priming or enhancing plant immunity against insect pests, which is a missed opportunity to exploit their ability to improve plant immunity, growth and nutrient acquisition.

Lastly, while *Actinobacteria* are well known in the literature for their potential as bioinsecticides and biopesticides more broadly, producing a wide range of specialized metabolites and other NPs with antimicrobial, antifungal and insecticidal properties, they represent a smaller proportion of the commercialized bioinsecticide products. Findings from this review indicate the opportunity exists to discover and produce valuable *Actinobacteria*-based leads with potential for new MoAs by broadening bioassay targets (including insect species screened and phenotypes captured), repurposing known NPs from other crop protection sectors, engineering strains for enhanced bioproduction or the generation of variants with improved potency, stability or safety profiles. As the demand for food security increases alongside increasingly stringent regulatory and consumer requirements for protecting the environment and ensuring food safety, *Actinobacteria*-based solutions present a valuable tool to support sustainable agriculture.

## Supplementary material

10.1099/mic.0.001690 Uncited Fig. S1.

## Abbreviations

*Bt*: *Bacillus thuringiensis*
GABA: gamma-aminobutyric acid
IPM: integrated pest management
IRAC: Insecticide Resistance Action Committee
JH: juvenile hormone
MoA: mode of action
nAChR: nicotinic acetylcholine receptor
NPs: natural products
NRP: non-ribosomal peptides
