# Supplementary material for: Exploring Actinobacteria for new insecticides and their delivery in crop protection
Source: Microbiology (Reading). 2026 Mar 24;172(3):001690. doi: 10.1099/mic.0.001690 (PMC13034444; doi:10.1099/mic.0.001690)
Supplement: Uncited Fig. S1. [file mic-172-01690-s001.pdf]

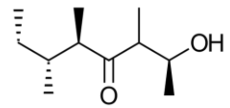

(2S,5R,6R)-2-hydroxy-3,5,6-trimethyloctan-4-one

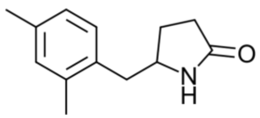

5-(2,4-dimethylbenzyl) pyrrolidin-2-one

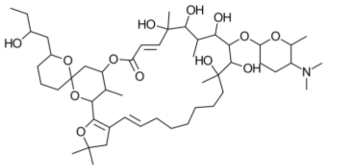

5'-epi-SPA-6952A

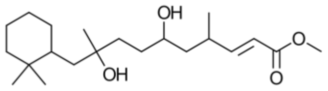

10-(2,2-dimethyl-cyclohexyl)-6,9-dihydroxy-4,9-dimethyl-dec-2-enoic acid methyl ester

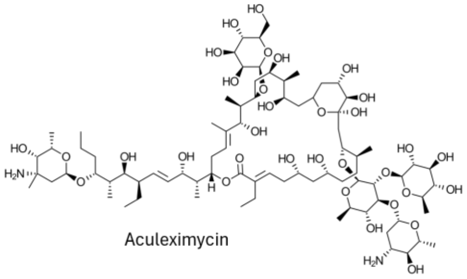

Aculeximycin

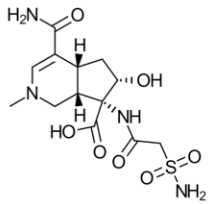

Altemicidin

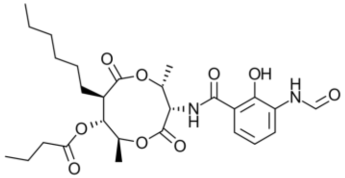

Antimycin A2

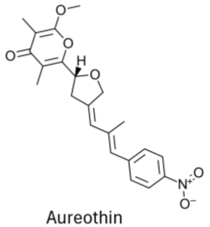

Aureothin

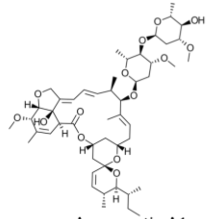

Avermectin A1a

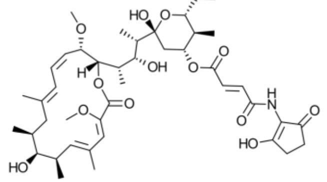

Bafilomycin B1

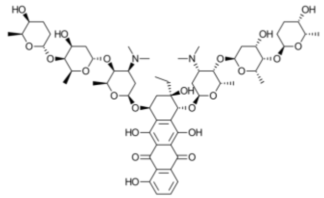

Cosmomycin

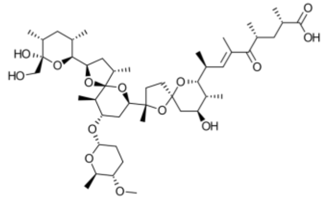

Dianemycin

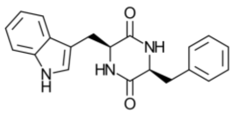

Diketopiperazine (cyclo-trp-phe)

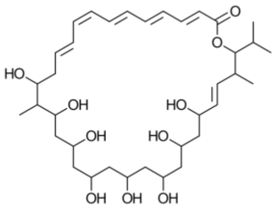

Faeriefungin

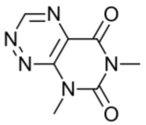

Fervenulin

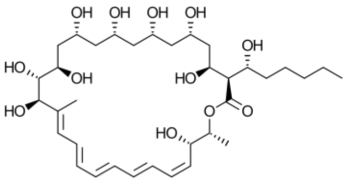

Fungichromin

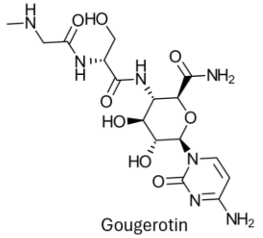

Gougerotin

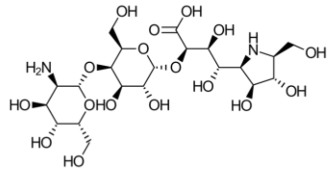

Gualamycin

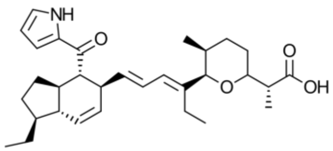

Indamycin

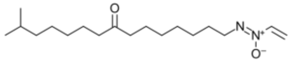

Jietacin A

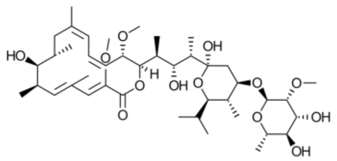

Leucanicidin

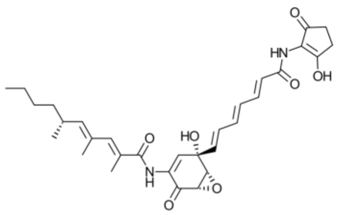

Manumycin

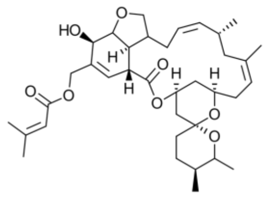

Meilingmycin

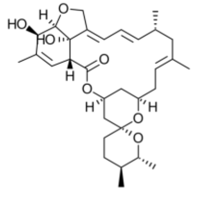

Milbemycin

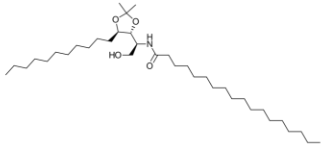

N-(1S-((4R, 5R)-2,2-dimethyl-5-undecyl-1,3-dioxolan-4-yl)-2-hydroxyethyl) stearamide

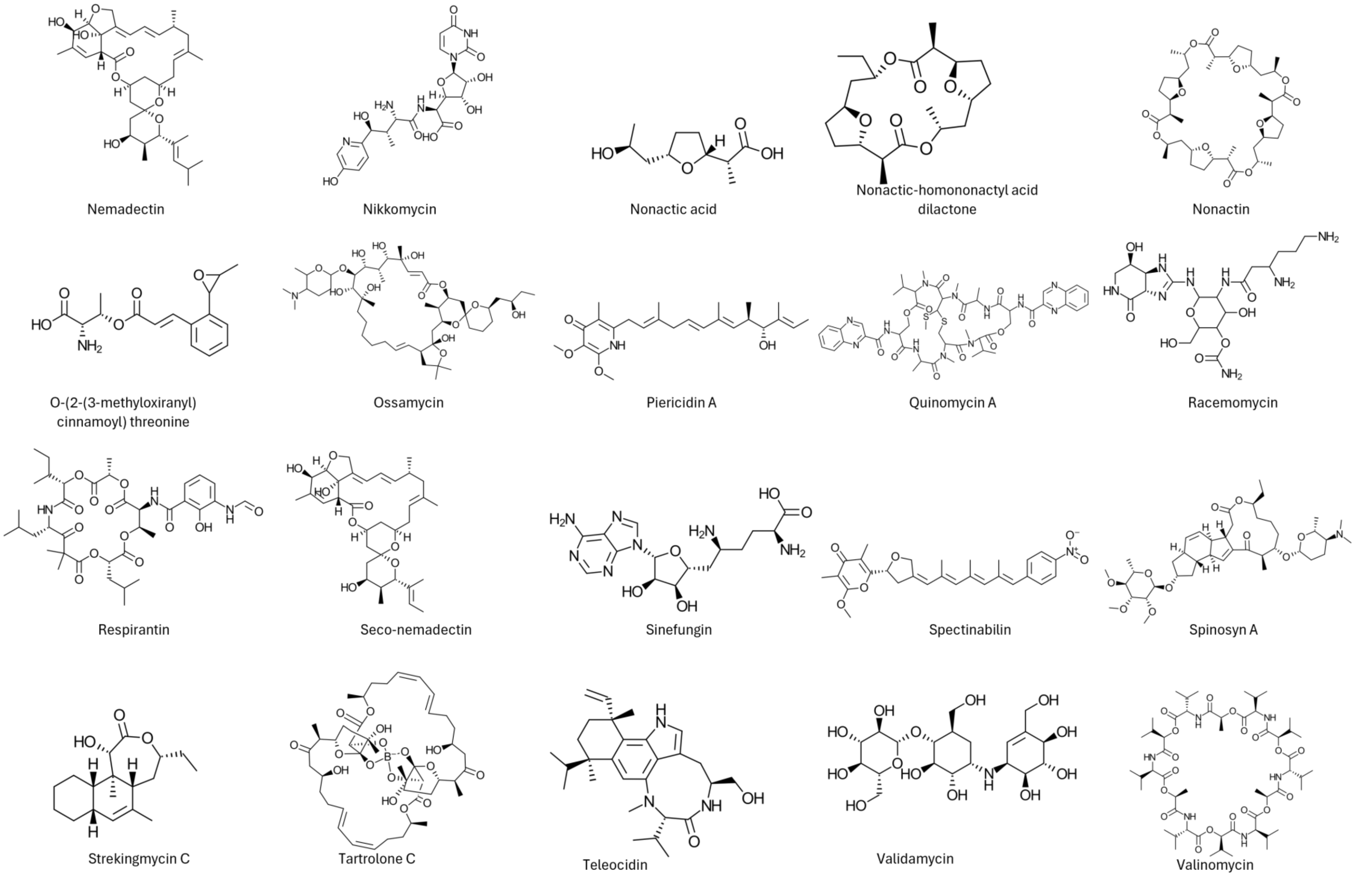

Supplementary Figure 1: Structures of reported insecticidal natural products from Actinobacteria, listed in alphabetic order. Stereochemistry is shown where available. A single congener is represented in cases where there are multiple known congeners for a certain natural product class (such as antimycin).
